# AB-stacked nanosheet-based hexagonal boron nitride

**DOI:** 10.1107/S2052520621000317

**Published:** 2021-03-17

**Authors:** Jae-Kap Lee, Jin-Gyu Kim, K. P. S. S. Hembram, Seunggun Yu, Sang-Gil Lee

**Affiliations:** a Center for Opto-Electronic Materials and Devices, Korea Institute of Science and Technology, Seoul, 02792, South Korea; b Division of Electron Microscopic Research, Korea Basic Science Institute, Daejeon, 34133, Republic of Korea; c Insulation Materials Research Center, Korea Electrotechnology Research Institute, Changwon, 51543, Republic of Korea

**Keywords:** hexagonal boron nitride, AB stacking, high-resolution transmission electron microscopy, simulation

## Abstract

Hexagonal boron nitride (*h*-BN) has been generally interpreted as having an AA stacking sequence. Evidence is presented in this article indicating that typical commercial *h*-BN platelets (∼10–500 nm in thickness) exhibit stacks of parallel nanosheets (∼10 nm in thickness) predominantly in the AB sequence. The AB-stacked nanosheet occurs as a metastable phase of *h*-BN resulting from the preferred texture and lateral growth of armchair (110) planes. It appears as an independent nanosheet or unit for *h*-BN platelets. The analysis is supported by simulation of thin AB films (2–20 layers), which explains the unique X-ray diffraction pattern of *h*-BN. With this analysis and the role of pressure in commercial high-pressure high-temperature sintering (driving nucleation and parallelizing the in-plane crystalline growth of the nuclei), a growth mechanism is proposed for 2D *h*-BN (on a substrate) as ‘substrate-induced 2D growth’, where the substrate plays the role of pressure.

## Abstract 2

The structure of *h*-BN (generally considered to have an AA stacking sequence) has been reinterpreted as AB stacking, based on the classical theory of crystal growth. A growth mechanism is proposed for commercial *h*-BN platelets as ‘pressure-induced 2D growth’ and for *h*-BN sheets on a substrate (in CVD or PVD approaches) as ‘substrate-induced 2D growth.

## 1. Introduction   

Boron nitride (BN) is comparable with graphite in its (layered) structure, as well as with respect to its physical properties (Wang *et al.*, 2017 ▸). The structures of its allotropes, *i.e.* soft ‘hexagonal’ (*h*-BN) and hard ‘cubic’ (*c*-BN), are comparable with those of graphite and diamond, respectively. The specific stacking structure of *h*-BN layers has not been clarified. It was reported initially to have an AB stacking sequence (Hassel, 1926 ▸), similar to Bernal AB graphite (Bernal, 1924 ▸). In 1950, Pease (1950 ▸) proposed AA stacking based on the calculated X-ray diffraction pattern (XRD) intensity, and this has generally been accepted as the structure of *h*-BN (Wang *et al.*, 2017 ▸; Pease, 1952 ▸; Topsakal *et al.*, 2009 ▸; Constantinescu *et al.*, 2013 ▸; Gilbert *et al.*, 2018 ▸; Alem *et al.*, 2009 ▸; Warner *et al.*, 2010 ▸). The analysis pointing to AA stacking is mostly based on energy calculations, where AA is more stable by approximately tens of meV compared to AB (Constantinescu *et al.*, 2013 ▸; Gilbert *et al.*, 2018 ▸; Liu *et al.*, 2003 ▸). The energy difference can lead the layers to slide even at room temperature by agitation. Liu *et al.* (2003 ▸) expected that real *h*-BN may have a mixture of AA and AB stacking based on the small energy difference of ∼0.012 meV.

Due to the presence of two elements, *i.e.* B and N, *h*-BN has two structural family groups of AB and AA, such as AB′ (B over B), A′B (N over N), AB (B over N) and AA (B over B), and AA′ (B over N) (Constantinescu *et al.*, 2013 ▸; Gilbert *et al.*, 2018 ▸). It is accepted that AA′ and AB′ are the minimum energy configurations in each group (Constantinescu *et al.*, 2013 ▸; Gilbert *et al.*, 2018 ▸); thus, AA and AB here refer to AA′ and AB′, respectively. Here, we also notice that the AA′ sequence of *h*-BN is not related to the AA′ sequence of graphite (Lee *et al.*, 2008 ▸, 2016 ▸). We also expect that the similarity of the X-ray diffraction (XRD) patterns for samples of *h*-BN with that for AA′ (2θ = 40–50°) is one reason for the analysis of the structure of BN as AA′ (like Pease’s analysis). Commercial *h*-BN reveals a unique XRD pattern (Yuan *et al.*, 2014 ▸; Li *et al.*, 2011 ▸; Huang *et al.*, 2000 ▸; Zhang *et al.*, 2017 ▸; Matović *et al.*, 2016 ▸), where the (100) peak at 2θ = 41.5° is stronger than the (101) peak at 2θ = 43.7°. Such relative intensity of the (100) peak with repsect to the (101) peak (which is a reverse of AB graphite) is observable for bulk AA′ *h*-BN (Fig. S1 in the supporting information), while the interplanar spacing of *h*-BN (3.34 Å) is close to the value of 3.35 Å for AB graphite (Bernal, 1924 ▸; Lee *et al.*, 2008 ▸).

With the recent focus on two-dimensional (2D) materials inspired by graphene (Novoselov *et al.*, 2004 ▸), understanding the structure of *h*-BN is vital to tune the band gap (Dai *et al.*, 2014 ▸; Ribeiro & Peres, 2011 ▸), as well as to design 2D *h*-BN-based heterostructures for electronic applications (Wang *et al.*, 2017 ▸; Behura *et al.*, 2015 ▸). Here we show that, unlike the conventional view, *h*-BN appears predominantly as an AB sequence, based on a series of simulations [energy, XRD and high-resolution transmission electron microscope (HRTEM)], as well as XRD and HRTEM analyses of commercial samples, and our study is consistent with the diverse data on the structure of *h*-BN reported in the literature.

## 2. Experimental   

**Figure 1:**
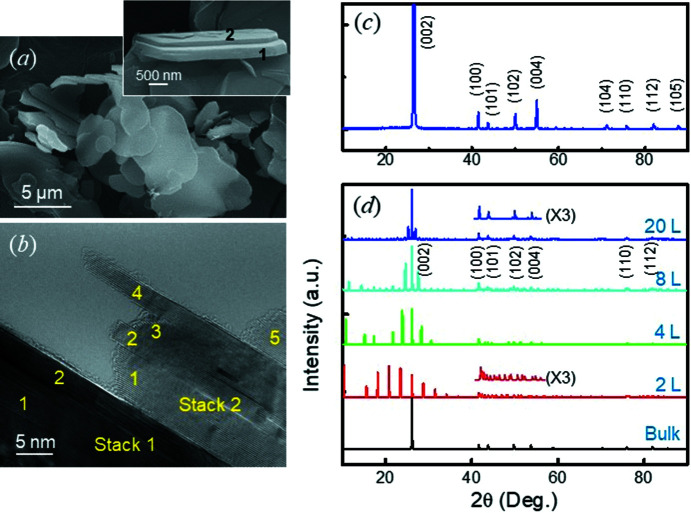
Analysis of *h*-BN samples. (*a*) A SEM image revealing the typical platelet shapes of commercial *h*-BN. The inset shows the overlap of two platelets (1 and 2), which reveals traces of sublayers (Fig. S2 in the supporting information). (*b*) A HRTEM image revealing stacks 1 and 2, corresponding to the two platelets shown in part (*a*), which are expected to be composed of at least two and five nanosheets, respectively. (*c*) An XRD pattern measured for the *h*-BN samples. (*d*) Simulated XRD patterns for the AB *h*-BN bulk and thin films with a different number of BN layers.

We analyzed commercial *h*-BN (3M Technical Ceramics, Germany), revealing the typical platelet shape with dimensions of micrometres. The samples were analyzed using an X-ray diffractometer (PANalytical X’pert Pro) with a Cu *K*α source, a scanning electron microscope (SEM) (Inspect F50, FEI) and two TEMs, *i.e.* Cs-corrected TEM (Libra 200 HT Mc, Carl Zeiss) and TitanTM80-300 (FEI). The selected area for the electron diffraction pattern (SAED) was acquired from a single (unstacked) BN sheet of approximately 10 nm in thickness (Figs. 1 ▸ and 2 ▸) to minimize the dynamical scattering effect. We used *CrystalMaker* (Palmer, 2014 ▸) to build atomic structures of *h*-BN for HRTEM and electron diffraction (ED) simulations, which were calculated using *JEMS* (CIME EPFL). Digital micrograph (Gatan Inc.) software was used to analyze the HRTEM images and their fast Fourier transform (FFT) patterns. In order to determine the suitable 1:1 combination of B and N, we scanned a wide range of their spectra. We considered the homogeneous distribution of B and N in a unit cell. The bilayer structure is considered by various stacking sequences. The suitable structure is obtained by observing the stability of various structures. The simulations were carried out by first-principles calculations implemented in *Quantum Espresso* (Giannozzi *et al.*, 2009 ▸). Ultra-soft pseudopotentials were used to represent interaction between ionic cores and valence electrons (Vanderbilt, 1990 ▸). Generalized gradient approximation was used for the exchange correlation energy of electrons (Perdew *et al.*, 1996 ▸). Plane wave basis with an energy cut-off of 40 Ry was used with a suitable mesh of grids (Monkhorst & Pack, 1976 ▸; Methfessel & Paxton, 1989 ▸). XRD patterns of all the structures were generated by the *FULLPROF* suite to provide a comparison with the experimental data (Rodríguez-Carvajal, 2001 ▸).

## 3. Results   

### 3.1. Structure analysis of *h*-BN platelets   

Fig. 1 ▸(*a*) shows the SEM image of commercial *h*-BN, synthesized by a high-pressure and high-temperature (HPHT) sintering process. The materials reveal the typical platelet shape where the dimensions vary widely in thickness (approximately hundreds of nm) and diameter (∼10 µm) (see also Fig. S2 in the supporting information). The samples also reveal the typical XRD pattern for *h*-BN (Fig. 1 ▸ *c*), where the relative intensity of the (100) peak at 2θ = 41.5° and of the (101) peak at 2θ = 43.7° [where the (100) peak is stronger than the (101) peak] (Yuan *et al.*, 2014 ▸; Li *et al.*, 2011 ▸; Huang *et al.*, 2000 ▸; Zhang *et al.*, 2017 ▸; Matović *et al.*, 2016 ▸). Such relative intensity is observable in the simulated pattern for AA′ BN (Fig. S1 in the supporting information). Our energy calculations show that AB′ and AA′ are stable phases in each structure group, and AA′ is the stable phase of *h*-BN (Fig. S3 in the supporting information). The results are consistent with those reported by others (Constantinescu *et al.*, 2013 ▸; Gilbert *et al.*, 2018 ▸; Liu *et al.*, 2003 ▸), where the energy differences are in the range of tens of meV. Hereafter, we refer to AA′ and AB′ simply as AA and AB, respectively.

**Figure 2:**
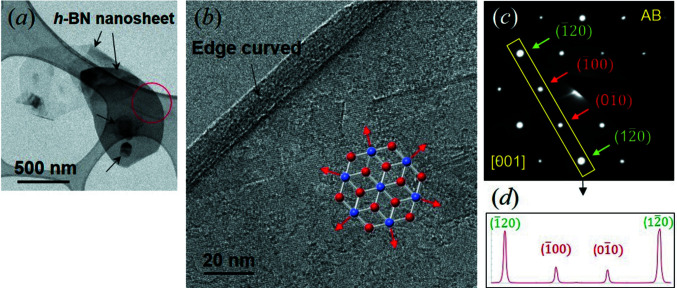
HRTEM images of *h*-BN nanosheets and SAED pattern analysis for a nanosheet. (*a*) A low magnification TEM image revealing several nanosheets (arrow) which appear to be overlapped. The red circle indicates the selected area of ∼400 nm in diameter where the ED pattern was acquired. (*b*) A HRTEM image of a nanosheet where the thickness is measured to be approximately 10 nm and an end is curved. The schematic in (*b*) depicts the texture growth of an AB *h*-BN nucleus. (*c*) A SAED pattern of the nanosheet. (*d*) An intensity profile of the spots in the rectangle in part (*c*). The intensity ratio of {120} to {100} spots is 3.3.

**Figure 3:**
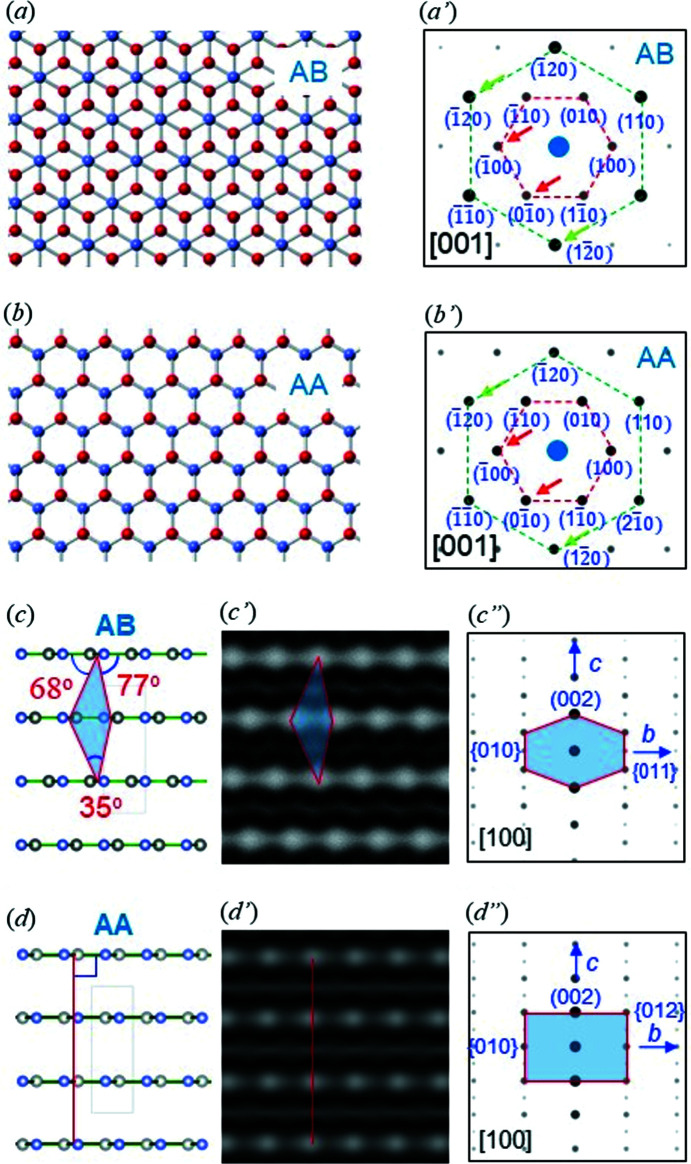
Simulated ED patterns. (*a*)/(*b*) Models for plane view AB and AA *h*-BN. (*a*′)/(*b*′) ED patterns for AB and AA *h*-BN, where the intensity ratios of the {120} and {100} spots are 2.8 and 0.95, respectively. (*c*)/(*d*) Models for the cross-sectional view of AB and AA *h*-BN. (*c*′)/(*d*′) Simulated HRTEM images for AB and AA *h*-BN. (*c*′′)/(*d*′′) Simulated ED patterns for AB and AA *h*-BN. AB reveals a hexagonal pattern (*c*′′), while AA reveals a rectangular pattern (*d"*).

An SAED pattern, measured from a sheet of approximately 10 nm in thickness (Fig. 2 ▸ *a*), is shown in Fig. 2 ▸(*b*), where the intensity of the {120} spots are much stronger than those of the {100} spots. The intensity ratio of the {120} and {100} spots measured from the intensity profile (Fig. 2 ▸ *d*) was 3.3, which is similar to that (2.8) of the simulation for the AB *h*-BN structure (Fig. 3 ▸ *a*′). The analysis demonstrates that the nanosheet is of an AB single-crystalline nature (Figs. 3 ▸ *a* and 3 ▸ *a*′). The samples (∼10 nm in thickness) measured here reveal evidence of AB *h*-BN (Fig. S4 in the supporting information). We could not identify SAED evidence for AA (Fig. 3 ▸ *d*). A SAED pattern for AB *h*-BN was reported by Gilbert *et al.* (2018 ▸), who deposited *h*-BN on iron foil by the chemical vapour deposition (CVD) method. On the other hand, the revelation of the edge of the sheet (Fig. 2 ▸ *b*) is due to the nature of the 2D structures where the ends appear as curved (Lee *et al.*, 2017 ▸).

**Figure 4:**
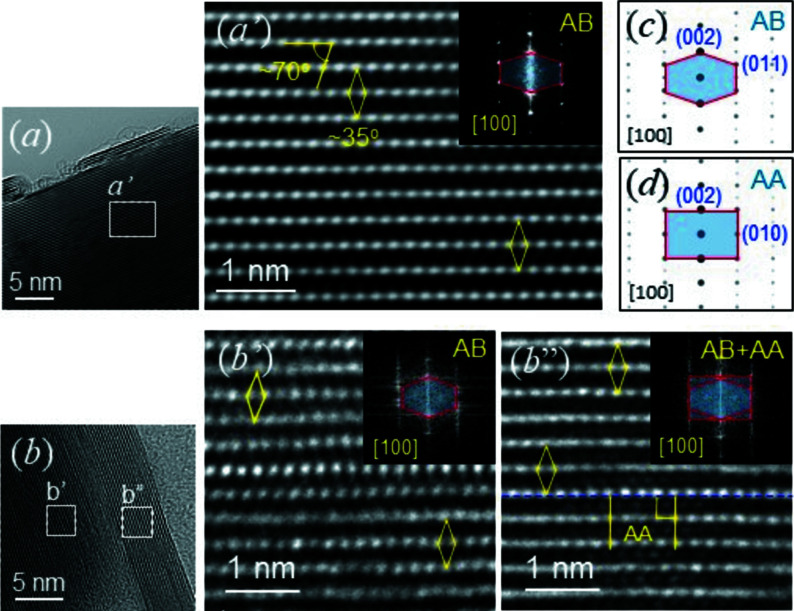
HRTEM images for *h*-BN samples. (*a*) A HRTEM image of a *h*-BN sample. (*a*′) Atomically resolved TEM image revealing the diagonal lattices of AB *h*-BN. (*b*) A HRTEM image of a *h*-BN sample. (*b*′)/(*b*′′) Atomically resolved TEM image for the rectangles in part (*b*), revealing the diagonal lattices for AB *h*-BN and the diagonal lattices for AB *h*-BN. The insets in parts (*a*′), (*b*′) and (*b*′′) indicate the FFT patterns obtained from each morphology. (*c*) Simulated ED pattern for AB *h*-BN. (*d*) Simulated ED pattern for AA *h*-BN.

The presence of AB *h*-BN is also evident in the cross-sectional HRTEM images shown in Fig. 4 ▸(*a*′) (see also Fig. S5 in the supporting information). White dots, due to each set of two atoms (B and N) on a cross-sectional HRTEM image for AB *h*-BN, form a ‘diagonal lattice’ with an angle of ∼35°, due to the vertical lines angled by ∼70° (exactly 67–75°; Fig. 3 ▸ *c*) with repsect to the horizontal direction. This explains the hexagonal fast Fourier transform (FFT) pattern for AB *h*-BN (see inset in Fig. 4 ▸ *a*′). Such a unique cross-sectional HRTEM morphology for AB was reported previously, and separately, by Tonkikh *et al.* (2016 ▸) and Sutter *et al.* (2013 ▸). Tonkikh *et al.* (2016 ▸) interpreted the lattices as AB, while Sutter *et al.* (2013 ▸) interpreted them as ABC rhombohedral BN (*r*-BN).

On the other hand, the sample shown in Fig. 4 ▸(*b*), is analyzed as an overlap of nanosheets. Indeed, many platelet *h*-BN samples reveal evidence of stacks comprising plural sheets of thickness ∼10 nm (Fig. 1 ▸ *b*). With the presence of the independent nanosheet (Fig. 2 ▸), the stack of nanosheets indicates that the nanosheet is a unit of a typical *h*-BN platelet. This shows that the samples should be analyzed as a nanosheet, *i.e.* a textured thin structure (Lee *et al.*, 2016 ▸). Simulated XRD patterns for thin AB *h*-BN, shown in Fig. 1 ▸(*d*), indicate that the (101) peak becomes weaker with an increasing number of layers, resulting in the unique relative intensity of *h*-BN (Fig. 1 ▸ *c*) (Yuan *et al.*, 2014 ▸; Li *et al.*, 2011 ▸; Huang *et al.*, 2000 ▸; Zhang *et al.*, 2017 ▸; Matović *et al.*, 2016 ▸), while textured AA *h*-BN reveals a rather strong (101) peak (Fig. S1 in the supporting information). The data indicate that the unique XRD pattern for *h*-BN (Fig. 1 ▸ *c*) is evidence of the AB nanosheet structure. The appearance of clear ED spots (Fig. 2 ▸ *b*) and FFT (see inset in Fig. 4 ▸ *a*) indicates that the nanosheets are of a well-developed single-crystalline nature.

### 3.2. Growth mechanism of *h*-BN   

**Figure 5:**
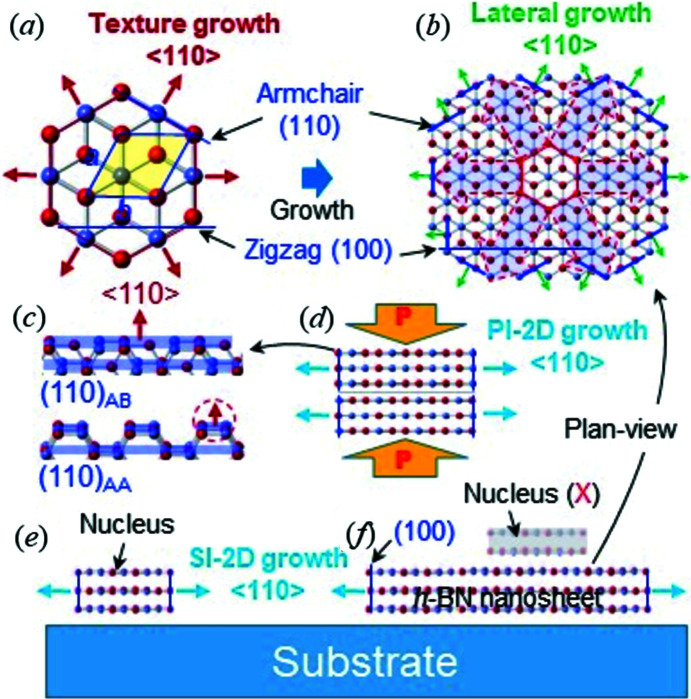
Growth mechanism of *h*-BN. (*a*) Explanation for the texture growth (red arrow) of an AB nucleus. (*b*) Explanation for the lateral growth (green arrow) of an AB nanosheet. (*c*) Schematic showing the (110)_AB_ and (110)_AA_ planes for three BN layers [translucent blue planes indicate (110)_AB_ or (110)_AA_]. The dashed circle indicates the localized growth of (110)_AA_ due to its localized arrangement of atoms. (*d*) Schematic explaining the PI-2D growth model of *h*-BN platelets in HPHT sintering. ‘P’ in big arrows indicates pressure. (*e*) Schematic explaining the SI-2D growth model of *h*-BN on a substrate. ‘X’ indicates the impossibility of the secondary nucleation onto the nanosheet.

Using the HRTEM and XRD data, we depict a formation mechanism for the *h*-BN nanosheet (Fig. 5 ▸). Due to the small energy difference, AA and AB nuclei can be formed at the initial stage of the synthesis (Fig. 5 ▸ *a*). Their growth is dominated by the armchair (011) plane with a higher surface energy of 5.5 J m^−2^ compared with 4.8 J m^−2^ for the zigzag (100) plane (the values are for graphite) (Abrahamson, 1973 ▸). This causes the preferred 〈110〉 texture growth of six directions on the nuclei (red arrow in Fig. 5 ▸ *a*). With the texture growth, many (local) lateral growths (also driven by 〈110〉) of the armchair (100) planes (green arrow in Fig. 5 ▸ *b*) result in the formation of the circular 2D nanosheet structure (Fig. 5 ▸ *b*), which explains the single-crystalline sheet shown in Fig. 2 ▸.

We infer that the crystalline lateral growth of the nanosheet (Fig. 5 ▸ *b*) occurs collectively, *i.e.* in the unit of the (110) plane. Here, the (110) plane of the AB structure, *i.e.* (110)_AB_ with a relatively uniform distribution of the atoms, can be effective in terms of the planar growth, compared with (110)_AA_, where atoms are localized (Fig. 5 ▸ *c*). We attribute the dominant existence of AB stacking to the relatively uniform distribution of the atoms in (110)_AB_, leading to the collective growth of (110)_AB_. In contrast, the growth of (110)_AA_ may occur at the unit of the localized atoms (red dots in Fig. 5 ▸ *c*), preventing its collective growth. We also expect that, in terms of planar growth, the zigzag edges of the (100) planes of AA or AB *h*-BN (Figs. 5 ▸ *a* and 5 ▸ *b*) may be less effective due to the uneven arrangement of the atoms. These infer that less stable AB appears typically as a metastable phase of *h*-BN. The analysis is supported by the in-plane HRTEM evidence of Li *et al.* (2011 ▸) for AB nanosheets (peeled from *h*-BN platelets), revealing the diagonal lattices unique to AB stacking (Lee *et al.*, 2016 ▸).

We paid attention to pressure in the commercial HPHT sintering process, which drives the nucleation of *h*-BN sheets. Under uniaxial pressure, vertical or angular growth of nuclei (against the direction of the applied pressure) might be prohibited. Such pressure-induced 2D (PI-2D) growth (Fig. 5 ▸ *d*) parallelizes the crystalline growth of the armchair (110) planes of the *h*-BN nuclei in the vessel of the system. This explains the laminated structure of the platelets, *i.e.* the in-plane stack of nanosheets shown in Figs. 1 ▸(*b*) and 4 ▸(*d*); a typical *h*-BN platelet is approximately hundreds of nm thick (Fig. 1 ▸ *a*). Here, the interface between the nanosheets can be disordered, *i.e.* twisted, resulting in relatively weak bonding.

The cleavage of commercial *h*-BN samples (along the basal plane of approximately hundreds of nm thick *h*-BN platelets into ∼10 nm thick nanosheets) with one-hour ball milling reported by Huang *et al.* (2000 ▸) supports our PI-2D growth model, although these authors explained the laminated decomposition as being due to Frank dislocation. This indicates that commercial *h*-BN is typically composed of nanosheets of ∼10 nm thickness (∼30 BN layers). The thickness of the *h*-BN sheets may be determined at the nucleation stage, depending on the driving force (pressure and temperature) applied to the system regardless of the sequence being AA or AB.

Our growth model for *h*-BN nanosheets, based on crystalline and PI-2D growth, can be extended to the 2D growth of *h*-BN on a substrate *via* CVD (Gilbert *et al.*, 2018 ▸; Behura *et al.*, 2015 ▸; Li *et al.*, 2019 ▸) and physical vapour deposition (PVD) (Tonkikh *et al.*, 2016 ▸; Sutter *et al.*, 2013 ▸) approaches. Indeed, CVD and PVD grown *h*-BN reveals a nanosheet structure of ∼5 nm in thickness or a faceted (triangular or hexagonal) morphology (Gilbert *et al.*, 2018 ▸; Li *et al.*, 2019 ▸). The latter can be explained by Wulff construction.

Thus, we propose a ‘substrate-induced 2D (SI-2D) model’ for the 2D *h*-BN growth on a substrate (Fig. 5 ▸ *d*). Here, the substrate plays the role of uniaxial pressure in the commercial HPHT process, driving heterogeneous nucleation, as well as guiding the 2D growth of the nuclei on a substrate with the unique crystalline growth of the armchair (110) planes. This SI-2D model supplements the previously suggested 2D growth models of *h*-BN (or graphene), including the layer-by-layer growth reported in the literature (Sutter *et al.*, 2013 ▸; Khan *et al.*, 2017 ▸). We expect that layer-by-layer growth or secondary nucleation onto *h*-BN nanosheets (Khan *et al.*, 2017 ▸) may not be active in reality because both need ‘collective’ (001) planar growth along the *c* axis and ‘coherent’ nucleation onto the in-plane of the nanosheet, respectively. Due to the stability of the basal plane (0.11 J m^−2^), which is not competitive with the prismatic planes (4.8–5.5 J m^−2^ in) (Abrahamson, 1973 ▸; Pierson, 1993 ▸), layer-by-layer growth or secondary nucleation may not occur in the 2D growth of *h*-BN. The analysis infers that the thickness of BN films in the CVD or PVD processes needs to be controlled at the stage of nucleation. We also infer that, in the SI-2D model, the number of BN layers (thickness) of nuclei (which grow laterally) may be affected by CVD or PVD conditions (pressure, temperature, *etc*.), rather than the condition of the substrate.

### 3.3. Co-existence of AB and AA stacking   

On the other hand, we could observe HRTEM morphological evidence for AA (vertical line lattice; Fig. 3 ▸ *d*), which appears with AB in a nanosheet (Fig. 4 ▸ *b*′′). Such co-existence of AA and AB, reported from mechanically milled commercial platelet samples (Huang *et al.*, 2000 ▸), confirms the results calculated by Liu *et al.* (2003 ▸). An independent AA nanosheet was included in the PVD samples of Sutter *et al.* (2013 ▸), together with another AB nanosheet. Thus, the appearance of AA is expected to be due to its crystalline growth (during sintering) and/or sliding of BN layers in preformed AB to form stable AA by agitation (after synthesis). For the latter case, the sliding of BN layers may depend on the state of a nanosheet, such as end curvature (Fig. 2 ▸ *b*), the presence of defects (dislocations or stacking faults) (Fig. S5 in the supporting information), as well as external factors, such as mechanical milling (Huang *et al.*, 2000 ▸) and temperature. The diffused FFT patterns shown in Figs. 4 ▸(*b*′) and 4(*b*′′) indicate that the BN layers are under strain. We attribute the strain to sliding of the BN layers to accommodate energy.

Our XRD analysis based on the simulations, providing general structural information, also supports our analysis of the structure of *h*-BN samples to be predominantly AB. The XRD simulations indicate that bilayer *h*-BN can reveal additional peaks in the range 2θ = 42–60° (Fig. 1 ▸ *d*) due to (enlarged) relaxation of their upmost layers. This may explain the appearance of the unexpected XRD peaks at 2θ = 42.6 and 45.6° reported by Yuan *et al.* (2014 ▸), who explained them as being due to *r*-BN. Indeed, their sample revealed a few *h*-BN layers, including mono- and bilayers, supporting our analysis of the XRD signal.

## 4. Summary   

We reinterpreted the structure of *h*-BN in terms of crystalline growth. Commercial *h*-BN platelets are composed of nanosheets in an AB stacking, resulting from the crystalline (texture and lateral) growth of the armchair (110) planes, as well as the PI-2D growth of AB nuclei. Stable AA stacking can appear locally. We propose a growth model for 2D *h*-BN in the CVD or PVD approaches to the crystalline growth on a substrate, leading to the SI-2D growth of BN nuclei. Our results, establishing the growth and resulting structure of *h*-BN, may provide a method of preparing an ideal 2D platform for electronics comparable with graphene structures. 

## Supplementary Material

Additional figures. DOI: 10.1107/S2052520621000317/rm5044sup1.pdf
