# Supplementary material for: AB-stacked nanosheet-based hexagonal boron nitride
Source: Acta Crystallogr B Struct Sci Cryst Eng Mater. 2021 Mar 17;77(Pt 2):260–5. doi: 10.1107/S2052520621000317 (PMC8040095; doi:10.1107/S2052520621000317)
Supplement: Supplementary file 1 [file b-77-00260-sup1.pdf]

STRUCTURAL SCIENCE
CRYSTAL ENGINEERING
MATERIALS

Volume 77 (2021)

Supporting information for article:

AB-stacked nanosheet-based hexagonal boron nitride

Jae-Kap Lee, Jin-Gyu Kim, K. P. S. S. Hembram, Seunggun Yu and Sang-Gil Lee

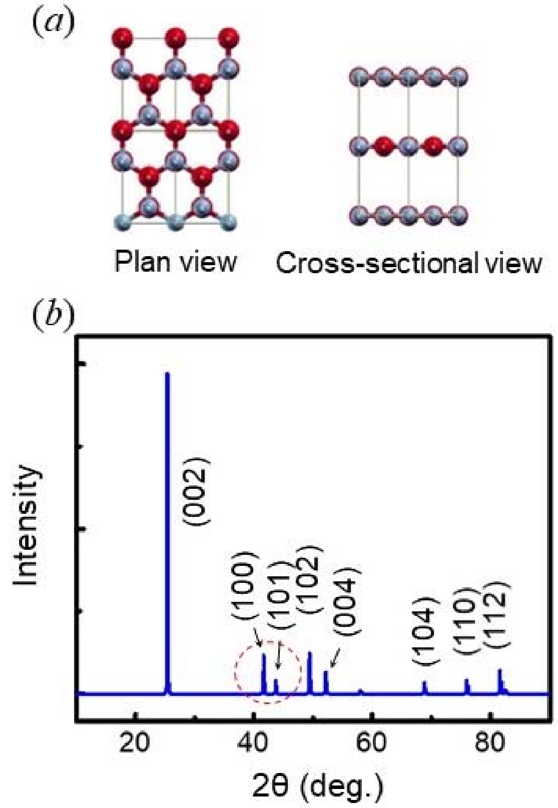

Fig. S1. Crystal structure and simulated XRD patterns for AA *h*-BN. (*a*) crystal unit for AA *h*-BN. (*b*) Simulated XRD pattern of *h*-BN bulk. The relative intensity of (100) and (101) for bulk is similar to the typical pattern for *h*-BN.

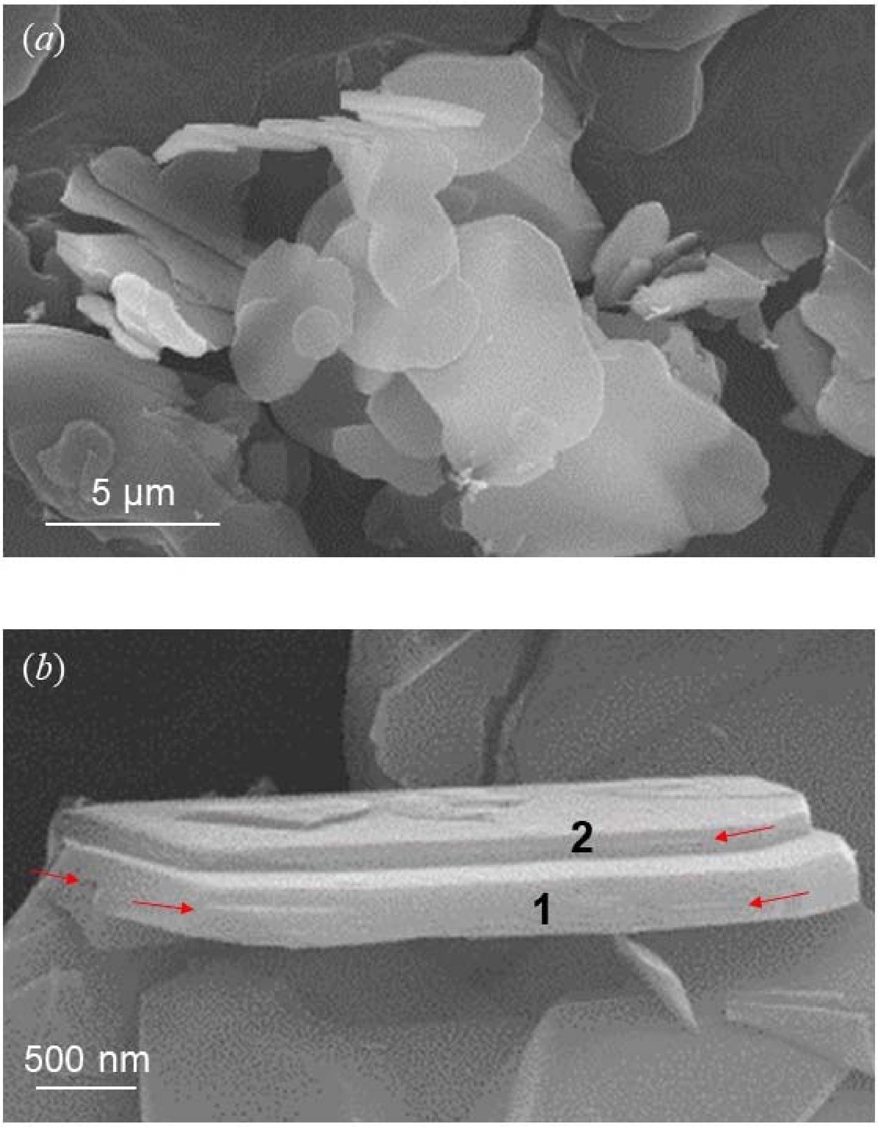

Fig. S2. SEM image of typical platelet *h*-BN. Overlapped two platelets (1 and 2) reveal the trace of layered structure (red arrows in (*b*)). We expect that each platelet is composed of many nanosheets (Fig. 1(*b*)).

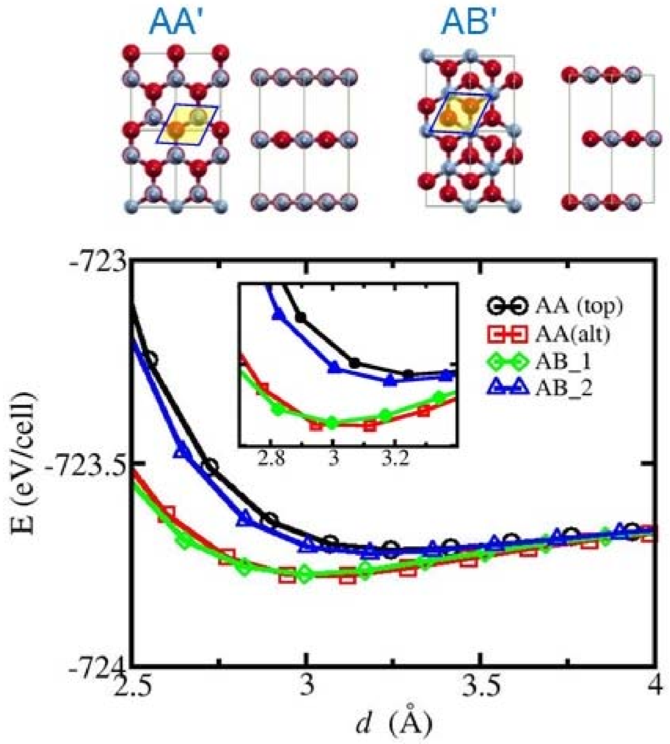

Fig. S3. Calculated stacking energy of bilayer *h*-BN. AB and AA' stackings reveal minimum energy configuration. Inset shows expanded graphs.

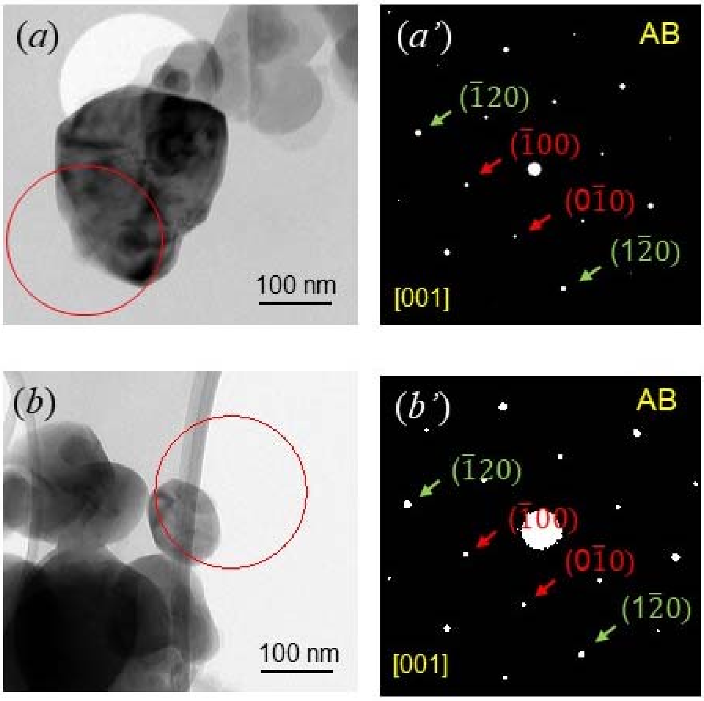

Fig. S4. TEM images and SAED patterns for *h*-BN nanosheet samples. Both patterns reveal evidence for AB stacking. Circle in (*a*) and (*b*) indicates the areas measured.

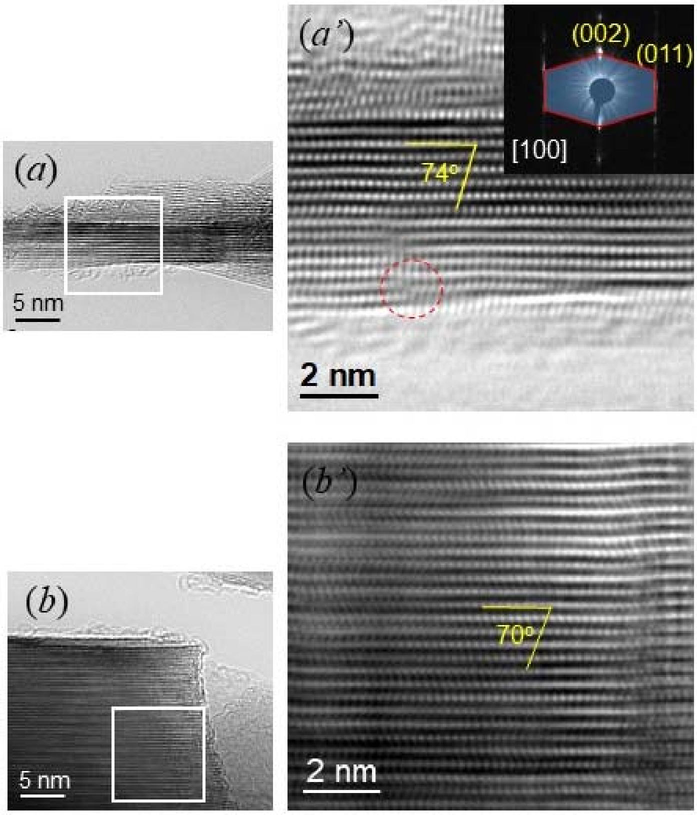

Fig. S5. Cross-sectional HRTEM images for *h*-BN samples. Both samples reveal evidence for AB stacking. (*a'*) and (*b'*) show the images for rectangles in (*a*) and (*b*), respectively. The red circle in (*a'*) indicates a stacking fault.
